# Non-Ligand-Induced Dimerization is Sufficient to Initiate the Signalling and Endocytosis of EGF Receptor

**DOI:** 10.3390/ijms17081200

**Published:** 2016-07-25

**Authors:** George Kourouniotis, Yi Wang, Steven Pennock, Xinmei Chen, Zhixiang Wang

**Affiliations:** Department of Medical Genetics and Signal Transduction Research Group, Faculty of Medicine and Dentistry, University of Alberta, Edmonton, AB T6G 2H7, Canada; kourouniotis.george@gmail.com (G.K.); yi.wang@cnl.ca (Y.W.); spennock76@gmail.com (S.P.); xinmei.chen@ualberta.ca (X.C.)

**Keywords:** EGF receptors, endocytosis, signal transduction, leucine zipper, dimerization

## Abstract

The binding of epidermal growth factor (EGF) to EGF receptor (EGFR) stimulates cell mitogenesis and survival through various signalling cascades. EGF also stimulates rapid EGFR endocytosis and its eventual degradation in lysosomes. The immediate events induced by ligand binding include receptor dimerization, activation of intrinsic tyrosine kinase and autophosphorylation. However, in spite of intensified efforts, the results regarding the roles of these events in EGFR signalling and internalization is still very controversial. In this study, we constructed a chimeric EGFR by replacing its extracellular domain with leucine zipper (LZ) and tagged a green fluorescent protein (GFP) at its C-terminus. We showed that the chimeric LZ-EGFR-GFP was constitutively dimerized. The LZ-EGFR-GFP dimer autophosphorylated each of its five well-defined C-terminal tyrosine residues as the ligand-induced EGFR dimer does. Phosphorylated LZ-EGFR-GFP was localized to both the plasma membrane and endosomes, suggesting it is capable of endocytosis. We also showed that LZ-EGFR-GFP activated major signalling proteins including Src homology collagen-like (Shc), extracellular signal-regulated kinase (ERK) and Akt. Moreover, LZ-EGFR-GFP was able to stimulate cell proliferation. These results indicate that non-ligand induced dimerization is sufficient to activate EGFR and initiate cell signalling and EGFR endocytosis. We conclude that receptor dimerization is a critical event in EGF-induced cell signalling and EGFR endocytosis.

## 1. Introduction

The epidermal growth factor (EGF) receptor (EGFR) (also named as HER1 and ErbB1) is a membrane receptor with intrinsic tyrosine kinase activity. EGFR is expressed in many cell types and regulates many cell functions [1,2]. EGFR is a 170 kDa membrane glycoprotein with three domains. The extracellular domain is heavily glycosylated with 622 amino acids, which is responsible for ligand binding and receptor dimerization. The transmembrane domain is an α-helical peptide of 23 amino acids. The 542-residue intracellular domain is composed of a conserved tyrosine kinase domain followed by a regulatory C-terminal tail. While EGFR signalling is critical for the control of many normal cell functions, the aberrant activity of EGFR by mutation and overexpression has played a key role in the origin and development of tumour cells [3,4,5].

Binding of EGF to EGFR stimulates cell mitogenesis and survival through various signalling cascades. EGF also stimulates rapid EGFR endocytosis into endosomes (EN) and its eventual degradation in lysosomes [1,2]. The immediate events induced by EGF binding include receptor dimerization, activation of intrinsic tyrosine kinase and autophosphorylation. The phosphorylated EGFR interacts with many signalling proteins, including Grb2, Shc, phospholipase C-γ1 (PLC-γ1), the p85 subunit of PI3K (p85), and Src, which initiates the activation of various signalling cascades [3,4,5]. For example, the interaction between EGFR and Shc/Grb2 results in the activation of Ras/ERK signalling pathways [2].

Clearly, the early events following EGF binding including receptor dimerization, kinase activation, autophosphorylation, and association with various binding proteins are essential for EGFR signalling and endocytosis. However, little is known whether ligand binding is required for all of these post-binding events or only required for dimerization, or whether dimerization is sufficient to stimulate kinase activation, autophosphorylation, and the binding of downstream proteins. It has been shown that inhibition of EGF-stimulated dimerization of EGFR does not impair receptor autophosphorylation or transmembrane signaling [6], which suggests that ligand-induced dimerization is not necessary for the activation of EGFR and downstream signalling. On the other hand, it has been reported that oncogenic ErbB2 is constitutively dimerized and permanently active [7,8,9]. Constitutively active ErbB2 homodimers lack the ability to bind ligand are much like ligand-activated EGFR in that they internalize rapidly and bind various downstream signalling proteins including Grb2 and Shc [10]. This suggests that ligand binding serves only to dimerize receptors, and that dimerization itself may mediate downstream events, such as recruitment of the receptor to coated pits and clathrin mediated endocytosis. Crystallographic investigation of EGFR implies that ligand binding induces the conformational change in its extracellular domain necessary to render EGFR competent for dimerization [11,12]. Mutant EGFR with the deletion of the dimerization loop weakly bind to EGF, but fails to be phosphorylated [11].

We have shown that the internalization of EGFR is controlled by EGFR dimerization, rather than the activation of EGFR kinase, and EGFR C-terminal sequences 1005–1017 and dileucine (LL) motif at 1010–1011 function as endocytic codes to mediate dimerization-driven EGFR endocytosis, independent of receptor kinase activity [13,14]. By using a controllable system to specifically form homodimers and heterodimers among ErbB receptors, we further showed that the heterodimer of ErbB2 and ErbB3 is deficient in endocytosis due to the lack of endocytic codes in their C-terminus. We also showed that two compatible sets of endocytic codes are essential for receptor endocytosis. Moreover, to mediate endocytosis, these two compatible sets of endocytic codes, each contained in one receptor molecule of the dimer, need be coordinated spatially [15]. Furthermore, we showed that dimerization of platelet-derived growth factor receptor (PDGFR) through its C-terminal fused FK506-binding protein (FKBP) induces PDGFR internalization [16].

However, the controllable system that we used dimerizes the receptors intracellularly, which is very different from ligand-induced dimerization. This difference may affect the status of the dimer resulting in a different EGFR endocytosis and downstream signalling. To overcome these potential problems, in this study we chose to artificially dimerize EGFR extracellularly and then examine the effects on EGFR endocytosis and activation, and the activation of signalling cascades downstream of EGFR. To this end, we examined whether EGFR is activated simply by dimerizing its transmembrane and cytoplasmic domains extracellularly via leucine zippers (LZ). A LZ is an α helix with leucine repeats (usually five residues long) spaced at every 7th position along its length. Due to the strong hydrophobic force of leucines, two complimentary zippers form high affinity dimers in solution. Certain transcription factors, such as c-Jun and c-Fos, contain LZs [17]. Recently, the dimerization of growth hormone receptor (GHR) was reported using LZs [18]. Based on these results, we replaced the EGFR extracellular domain with a c-Fos LZ domain and tagged this chimeric receptor with EGFP. We show that chimeric LZ-EGFR-GFP is constitutively dimerized and autophosphorylated. In a manner similar to ligand-induced EGFR phosphorylation, LZ-EGFR is phosphorylated at all five of its principle C-terminal tyrosines. The phosphorylated LZ-EGFR is localized at both the plasma membrane and endosomes, suggesting it is capable of being endocytosed. Moreover, LZ-EGFR activates signalling pathways involving Shc ERK and Akt. These signals are physiologically potent, eventually leading to cell proliferation.

## 2. Results

### 2.1. Expression and Dimerization of Leucine Zipper (LZ)-Epidermal Growth Factor Receptor (EGFR)-Green Fluorescent Protein (GFP)

**Figure 1:**
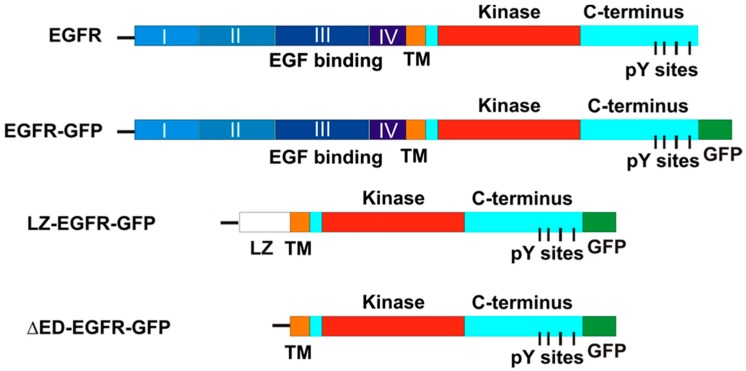
Schematic illustration of EGFR-GFP, LZ-EGFR-GFP and ΔED-(extracellular-domain deleted)-EGFR-GFP as compared to wild type EGFR (wtEGFR). The chimeric construct LZ-EGFR-GFP started with the signal sequence of wtEGFR (line), followed by a LZ sequence that replaced the complete extracellular domain of EGFR. ΔED-EGFR-GFP is a truncated EGFR with the deletion of its entire extracellular domain. The green fluorescent protein (GFP) is tagged to the C-terminus of EGFR.

Several EGFR plasmids were constructed (described in Materials and Methods) as shown in Figure 1.

**Figure 2:**
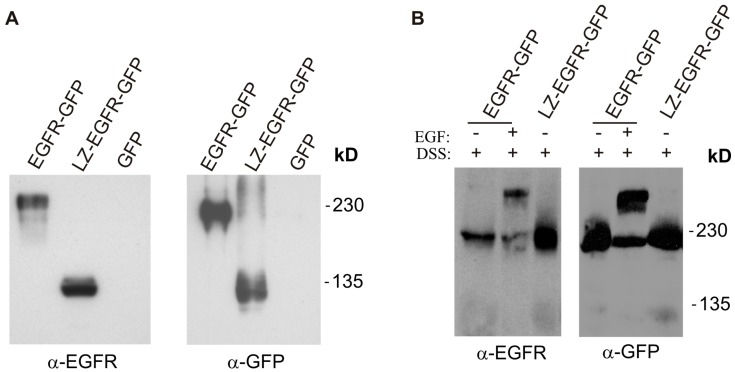

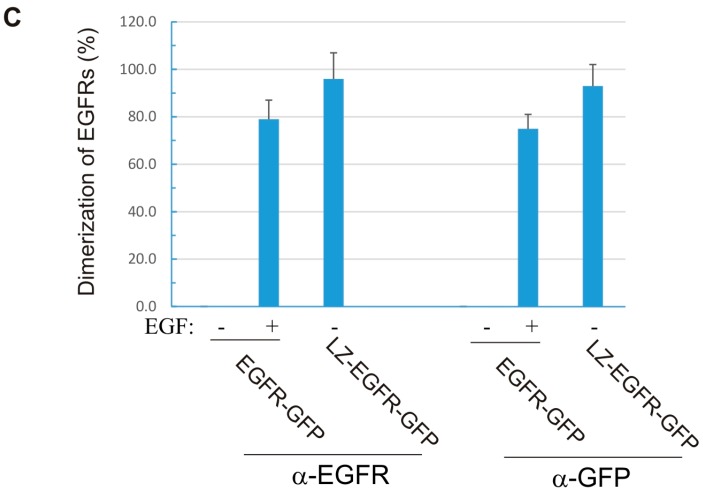
Expression and dimerization of LZ-EGFR-GFP. 293T cells were transiently transfected with LZ-EGFR-GFP, EGFR-GFP or empty vector (GFP). (**A**) EGFR-GFP and LZ-EGFR-GFP were visualized by immunoblotting of the total lysates with antibodies to either EGFR or GFP; (**B**) Cells were crosslinked with disulfosuccinimidyl suberate (DSS). Both LZ-EGFR-GFP monomer (105 kD) and dimer (210 kD) were visible in the resulting immunoblots. EGFR-GFP dimerized only after EGF stimulation, as expected; (**C**) Quantification of the data from (**B**). The band is quantitated by densitometry with image J software (National Institute of Health, Bethesda, MD, USA) and the receptor dimerization was expressed as the percentage of dimers among the total receptor proteins. Each value is the mean of at least three independent experiments and the error bar represents the standard error.

To determine whether the introduction of a LZ into EGFR results in constitutive dimerization of EGFR transmembrane and intracellular domains, we transiently transfected 293T cells with a plasmid encoding LZ-EGFR-GFP. 293T cells transfected with EGFR-GFP were used as a control. Immunoblotting of the total lysates with anti-EGFR and anti-GFP antibodies showed the presence of a strong band at 105 kD for cells transfected with LZ-EGFR-GFP, and at 210 kD for cells transfected with EGFR-GFP (Figure 2A). This indicates both LZ-EGF-GFP and EGFR-GFP were well expressed with the expected molecular weight.

To determine whether the introduction of a LZ results in the dimerization of EGFR, 293T cells transiently transfected with LZ-EGFR-GFP or EGFR-GFP were crosslinked with disulfosuccinimidyl suberate (DSS). Immunoblotting with anti-EGFR and anti-GFP antibodies revealed that most of the LZ-EGFR-GFP dimerized (Figure 2B,C). As a positive control, we showed that EGFR-GFP dimerized following EGF stimulation as expected (Figure 2B,C).

### 2.2. Subcellular Distribution of LZ-EGFR-GFP

**Figure 3:**
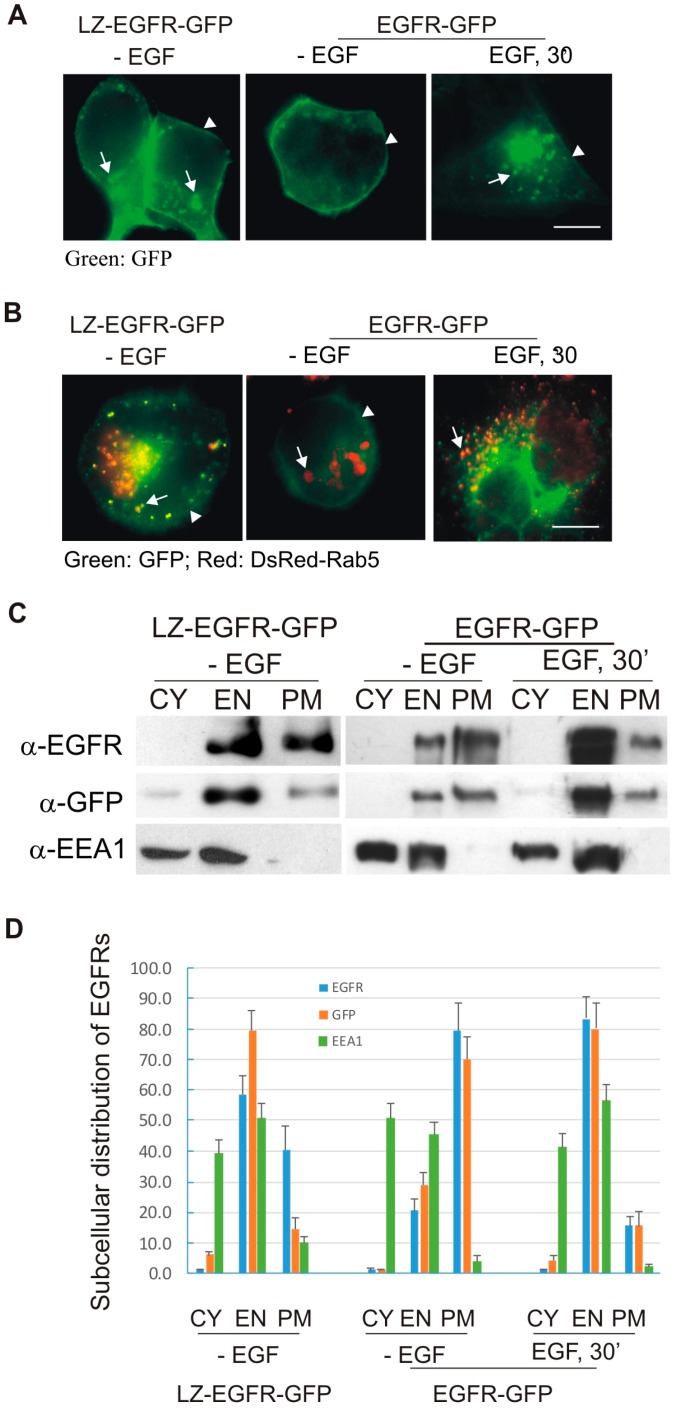
Subcellular distribution of LZ-EGFR-GFP. (**A**,**B**) Fluorescence analysis of subcellular EGFR localization. 293T cells were transiently transfected with LZ-EGFR-GFP or EGFR-GFP (**A**), or co-transfected with DsRed-Rab5 and LZ-EGFR-GFP or EGFR-GFP (**B**). The cells were treated with or without EGF. The subcellular localization of EGFR and Rab5 was revealed by the intrinsic fluorescence of GFP and DsRed. Co-localization of EGFR and Rab5 was indicated by yellow. Arrows denote endosomes and arrowheads denote plasma membrane regions. Size bar = 20 µm; (**C**) Subcellular fractionation and immunoblotting analysis. 293T cells transiently expressing LZ-EGFR-GFP were homogenized and subcellularly fractionated into cytoplasmic (CY), endosomal (EN) and plasma membrane (PM) fractions, confirmed by immunobloting the cooresponding fraction lysates with antibodies to EGFR, GFP and early endosome antigen 1 (EEA-1), respectively. 293T cells transfected with EGFR-GFP were used as controls; (**D**) Quantification of the data from (**C**). Bands were quantitated by densitometry with image J software and subcellular distribution of the proteins among the three fractions (CY, EN, and PM) was expressed as percentage of the total protein content of all three fractions combined.Each value is the average of at least three independent experiments and the error bar is the standard error.

We next determined the subcellular distribution of LZ-EGFR-GFP by fluorescence microscopy. 293T cells were transfected with LZ-EGFR-GFP, and cellular localization was determined by visualizing the intrinsic fluorescence of EGFP. 293T cells transfected with EGFR-GFP were used as the control. In all cases, cells were deprived of serum for 24 h prior to treatment/fixation to ensure that most receptors were coordinated at the plasma membrane. As shown in Figure 3A, consistent with previous findings, EGFR-GFP was localized to the plasma membrane without EGF, and then to endosomes following EGF stimulation for 30 min. LZ-EGFR-GFP, however, was localized at both the plasma membrane and intracellularly on a sub-population of vesicular structures indicative of endosomes (Figure 3A); this occurred in the absence of EGF, which is not bound by this chimeric protein in any case.. To demonstrate the localization of LZ-EGFR-GFP in endosomes, 293T cells were co-transfected with LZ-EGFR-GFP and DsRed-tagged wtRab5 (Figure 3B). A portion of LZ-EGFR-GFP co-localized with DsRed-Rab5, as shown by yellow vesicular structures, indicating that a portion of LZ-EGFR-GFP was localized to Rab5-positive endosomes. In control cells co-transfected with EGFR-GFP and DsRed-Rab5, the receptor co-localized with DsRed-Rab5 following 30 min of EGF stimulation, as expected (Figure 3B). These results suggest that a portion of LZ-EGFR-GFP is being constitutively endocytosed from the plasma membrane to endosomes, independent of ligand.

The plasma membrane and endosome localization of LZ-EGFR-GFP was further analyzed by subcellular fractionation. Immunoblotting demonstrated that LZ-EGFR-GFP localized to both the plasma membrane and endosome fractions (Figure 3C,D). The early endosome antigen 1 (EEA1) was used as a marker for endosomes. As a control, wild type EGFR, pre-localized to the plasma membrane through serum-deprivation, localized to the endosomes following EGF stimulation for 30 min.

### 2.3. Kinase Activation and Phosphorylation of LZ-EGFR-GFP

**Figure 4:**
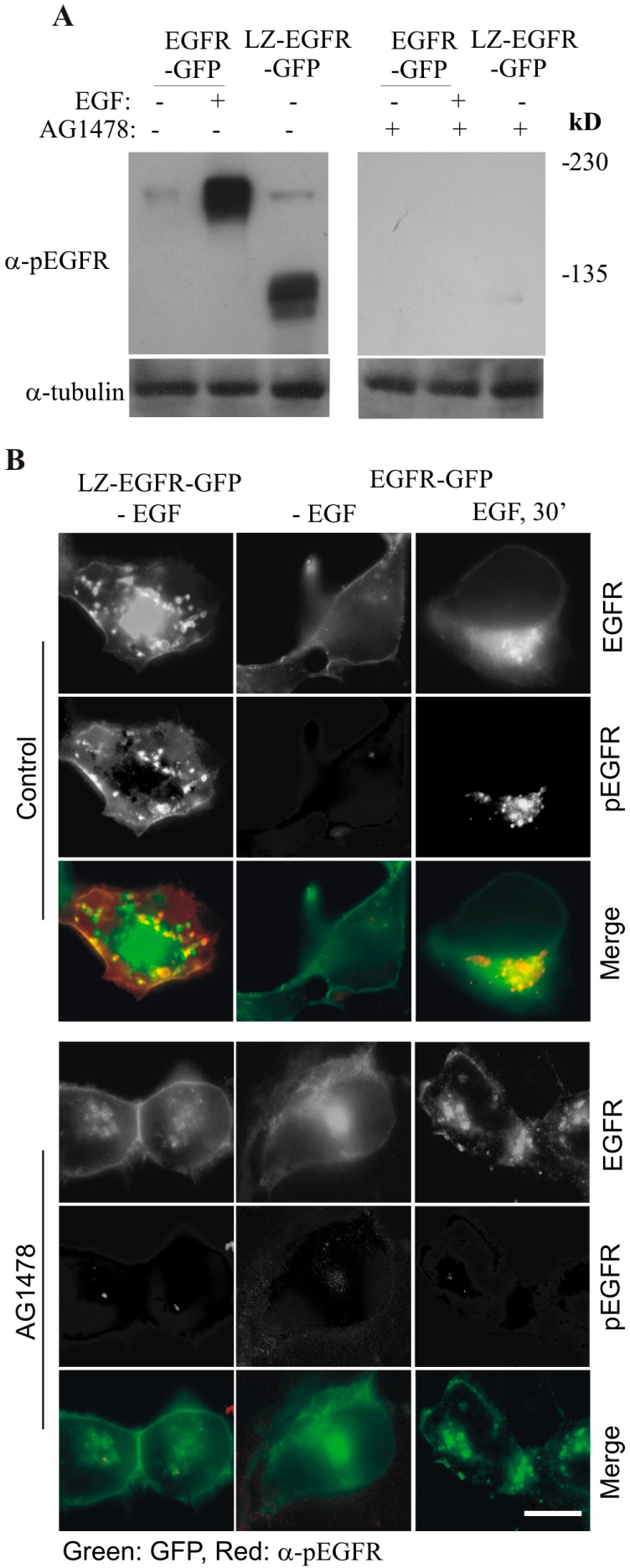
Phosphorylation of LZ-EGFR-GFP and the dependence on intrinsic tyrosine kinase activity. (**A**) Immunoblotting. 293T cells were transiently transfected with EGFR-GFP or LZ-EGFR-GFP. The cells were serum starved for 24 h and then were treated with EGF and/or AG1478 as indicated. Cell lysates were subjected to immunoblotting analysis with mouse anti-pEGFR antibody; (**B**) Immunofluorescence. 293T cells transiently transfected with either LZ-EGFR-GFP or EGFR-GFP were serum starved for 24 h. The cells were then treated with EGF and/or AG1478 as indicated. EGFR phosphorylation was examined by anti-pEGFR antibody followed by the secondary antibody conjugated with TRITC. Co-localization (yellow) of LZ-EGFR-GFP or EGFR-GFP (green) with p-EGFR (red) was determined by indirect immunofluorescence. Size bar = 20 µm.

We next determined whether dimerization of EGFR by LZ resulted in the activation (phosphorylation) of the receptor. As shown in Figure 4A, immunoblotting with anti-phospho-EGFR (pEGFR) antibody revealed that both dimer and monomer of LZ-EGFR-GFP were phosphorylated. To demonstrate that this phosphorylation is induced by the intrinsic kinase activity of EGFR, we blocked EGFR kinase activity with the EGFR tyrosine kinase inhibitor AG1478 and examined the phosphorylation status of LZ-EGFR-GFP. We showed that the phosphorylation of LZ-EGFR-GFP was blocked following treatment with AG1478 (Figure 4A). This indicates that LZ-EGFR-GFP is phosphorylated by its intrinsic tyrosine kinase activity alone.

This finding was further supported by indirect immunofluorescence. 293T cells were transiently transfected with LZ-EGFR-GFP or EGFR-GFP and subjected to indirect immunofluorescence with antibody to pEGFR followed with a secondary antibody conjugated with Tetramethylrhodamine (TRITC) (Figure 4B). We showed that LZ-EGFR-GFP was phosphorylated and localized to both the plasma membrane and endosomes as indicated in yellow. As expected for our control experiment, EGFR-GFP was not phosphorylated and localized to the plasma membrane prior to EGF stimulation. Following EGF stimulation for 30 min, however, the receptor was phosphorylated and internalized into endosomes. Moreover, inhibition of EGFR kinase activity by AG1478 blocked phosphorylation of both LZ-EGFR-GFP and EGFR-GFP (Figure 4B). This reinforces our finding that phosphorylation of LZ-EGFR-GFP is due to its intrinsic kinase activity. It should be noted, however, that inhibition of EGFR kinase activation by AG1478 did not inhibit the endosome localization of LZ-EGFR-GFP, consistent with our previous finding that EGFR kinase activation is not required for its internalization [19].

**Figure 5:**
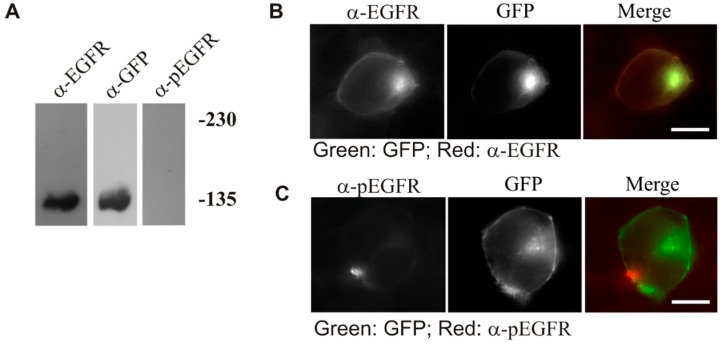
Expression, phosphorylation and subcellular localization of ΔED-EGFR-GFP. 293T cells were transiently transfected with ΔED-EGFR-GFP. (**A**) The expression and phosphorylation of ΔED-EGFR-GFP. Following the transfection for 48 h, the cell were lysed and total cell lysates were used to determine the expression and phosphorylation of ΔED-EGFR-GFP by immunoblotting; (**B**) Subcellular distribution of ΔED-EGFR-GFP. Following the transfection for 48 h, the subcellular localization of ΔED-EGFR-GFP was revealed by the intrinsic GFP and by anti-EGFR antibody followed by TRITC-conjugated secondary antibody; (**C**) The phosphorylation of ΔED-EGFR-GFP. Following the transfection for 48 h, the phosphorylation of ΔED-EGFR-GFP was revealed by anti-pEGFR antibody followed by TRITC-conjugated secondary antibody. Size bar = 20 µm.

To eliminate the possibility that the observed activation of LZ-EGFR-GFP is due the introduction of LZ rather than the deletion of EGFR extracellular domain, we deleted LZ from LZ-EGFR-GFP and transfected 293T cells with this mutant (ΔED-EGFR-GFP). We showed that ΔED-EGFR-GFP is not phosphorylated and is only localized at the plasma membrane (Figure 5). This indicates that deletion of EGFR extracellular domain itself is not sufficient to activate EGFR and stimulate its endocytosis.

Together, our results indicate that fusion of a LZ with the transmembrane and intracellular domain of EGFR leads to the dimerization of this chimeric receptor. Such dimerization then results in constitutive activation of intrinsic tyrosine kinase function and the subsequent phosphorylation of carboxyl terminal tyrosine residues.

### 2.4. LZ-EGFR-GFP Is Fully Activated

**Figure 6:**
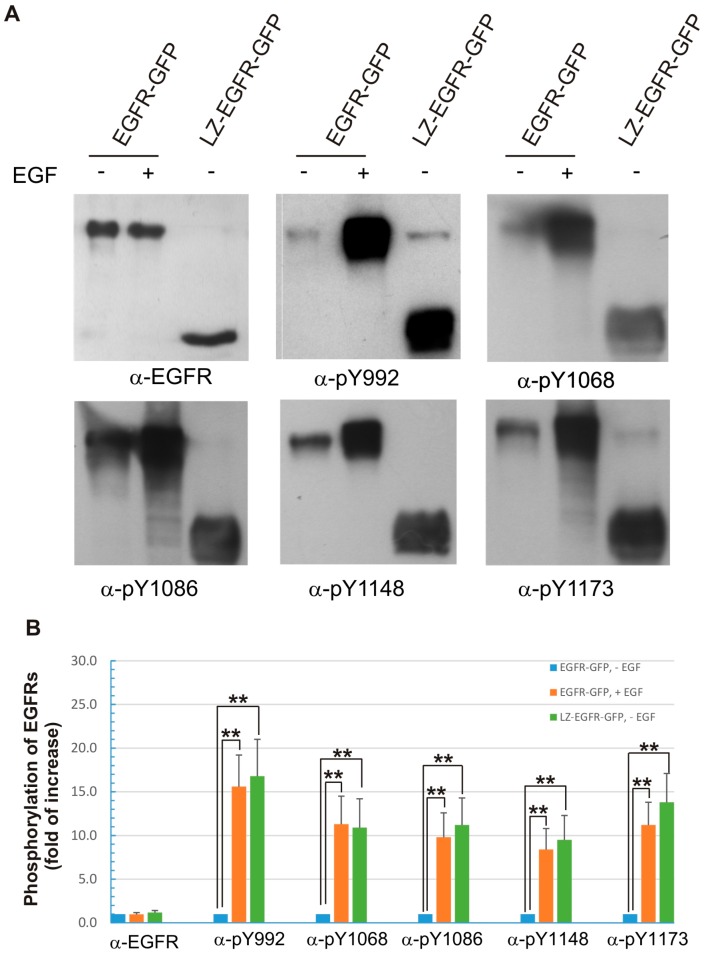
Phosphorylation of the five major C-terminal tyrosine residues of EGFR-GFP and LZ-EGFR-GFP. (**A**) 293T cells were transiently transfected with EGFR-GFP or LZ-EGFR-GFP. Following serum starvation for 24 h, cells were treated with or without EGF. The cell lysates were subjected to immunoblotting analysis with rabbit anti-pEGFR (pY992), anti-pEGFR (pY1068), anti-pEGFR (pY1086), anti-pEGFR (pY1148) and anti-pEGFR (pY1173) antibodies; (**B**) Quantification of the data from (**A**). The band is quantitated by densitometry with image J software. The phosphorylation level of the control (EGFR-GFP, without EGF treatment) was set to 1 and the phosphorylation of the receptors under other conditions was expressed as the fold increase compared to control. Each value is the average of at least three independent experiments and the error bar is the standard error. **: *p* < 0.01.

We next compared the specific tyrosine phosphorylation status of LZ-EGFR-GFP and EGF-activated EGFR-GFP. It is well established that EGF induces the phosphorylation of multiple tyrosine residues at the C-terminus of EGFR. These tyrosine residues include Y992, Y1068, Y1086, Y1148 and Y1173. Indeed, immunoblotting of 293T cells transfected with EGFR-GFP with these five phosphotyrosine-specific antibodies revealed that all of them were activated in response to EGF stimulation. Similarly, all five tyrosine residues were phosphorylated in LZ-EGFR-GFP without EGF stimulation (Figure 6). Despite the use of SDS in our gels, a small fraction of the LZ-EGFR remained dimerized, attributable to the strength of the di-LZ association. Though the phosphorylation of LZ-EGFR dimers at tyrosine residues 1068, 1086, and 1148 were very weak, this is due to differing sensitivities of our phospho-specific EGFR, and overexposure of these membranes reveals the activated dimer (data not shown). These results indicate that constitutive dimerization induced by LZ fully activates EGFR tyrosine kinase and phosphorylates its C-terminal tyrosine residues.

### 2.5. Activation of Various Signalling Pathways by LZ-EGFR-GFP

**Figure 7:**
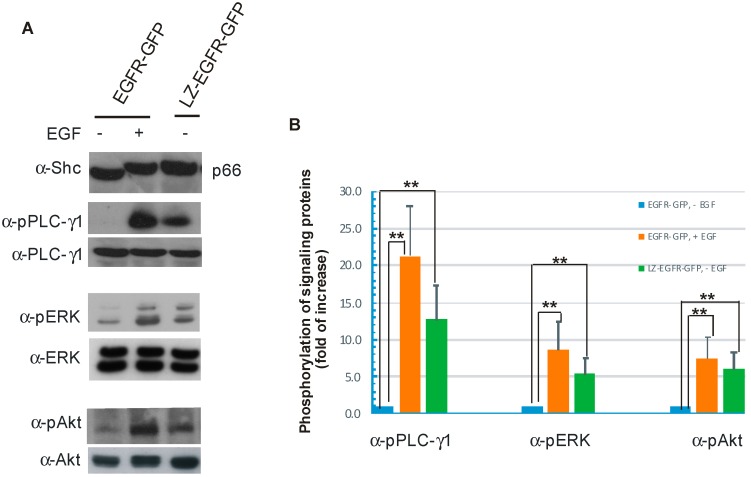
Stimulation of various signal transduction pathways by activation of EGFR-GFP or LZ-EGFR-GFP. (**A**) 293T cells were transiently transfected with EGFR-GFP or LZ-EGFR-GFP. Following serum starvation for 24 h, cells were treated with or without EGF. The cell lysates were subjected to immunoblotting analysis with rabbit anti-SHC, rabbit anti-phospho-PLC-γ1, rabbit anti-PLC-γ1, mouse anti-phospho-ERK1/2, mouse anti-Erk1/2, rabbit anti-phospho-Akt and rabbit anti-Akt antibodies; (**B**) Quantification of the data from (**A**). The band is quantitated by densitometry with image J software. The protein phosphorylation level of the control (EGFR-GFP, without EGF treatment) was set to 1 and the phosphorylation of the proteins under other conditions was expressed as the fold increase compared to control. Each value is the average of at least three independent experiments and the error bar is the standard error. **: *p* < 0.01.

These five phosphorylated tyrosine residues have been shown to bind and subsequently activate numerous signalling proteins/pathways including Grb2/Shc/Ras/ERK, PI3K/Akt, and PLC-γ1 pathways [5]. We next determined the phosphorylation status of these signalling proteins in 293T cells transiently transfected with LZ-EGFR-GFP. As shown in Figure 7A, control cells transfected with EGFR-GFP and stimulated with EGF resulted in the up-shift of the Shc p66 isoform, marking it as phosphorylated. A similar up-shift of the Shc p66 isoform was observed for the cells transiently transfected with LZ-EGFR-GFP, indicating that constitutive activation of LZ-EGFR results in the activation of Shc.

We next determined whether constitutively activated LZ-EGFR stimulated downstream signalling proteins including PLC-γ1, ERK, and Akt by immunoblotting with antibodies specific to phosphorylated PLC-γ1, ERK, and Akt. We showed that constitutively activated LZ-EGFR, and EGF-activated EGFR, phosphorylated PLC-γ1, ERK, and Akt (Figure 7A,B). These data indicate that non-ligand-induced dimerization of EGFR through LZ is sufficient to activate the major signalling pathways critical for various cell functions including mitogenesis and anti-apoptosis. It is interesting to note that even though the LZ-EGFR dimer is constitutively active, and leads to significant stimulation of downstream proteins, EGF induces a more robust phosphorylation of these factors.

### 2.6. Stimulation of Cell Proliferation by LZ-EGFR

**Figure 8:**
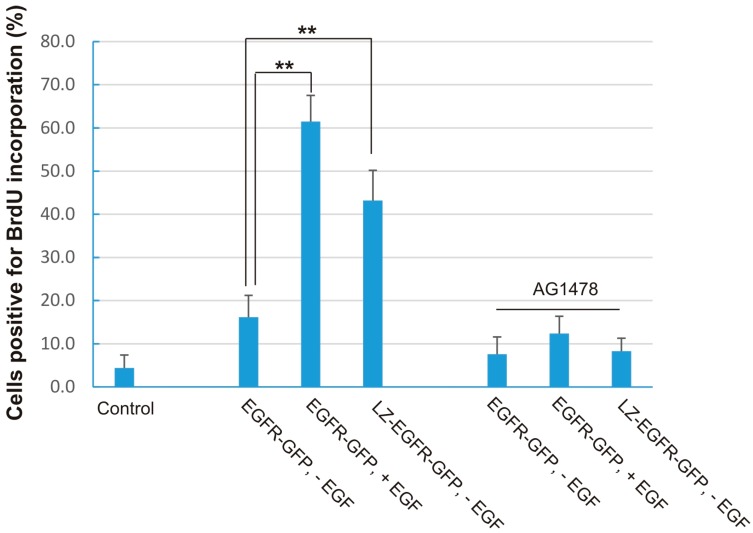
Stimulation of DNA synthesis by LZ-EGFR-GFP. 293T cells were transiently transfected with EGFR-GFP or LZ-EGFR-GFP. Following serum starvation for 24 h, cells were treated with EGF and/or AG1478 as indicated. DNA synthesis was determined by BrdU incorporation as described in the Materials and methods. Cells were counted at 300 per sample and data was plotted as the mean of at least three experiments. The error bar is the standard error. **: *p* < 0.01.

Since constitutively activated LZ-EGFR-GFP stimulates numerous signalling proteins implicated in cell mitogenesis, we determined whether LZ-EGFR-GFP induced cell proliferation. 293T cells transiently transfected with EGFR-GFP or LZ-EGFR-GFP were analyzed for cell proliferation using Bromodeoxyuridine (BrdU) incorporation experiments. As shown in Figure 8 in cells expressing EGFR-GFP, EGF stimulates strong BrdU incorporation (61%), whereas in the absence of EGF stimulation the BrdU incorporation rate is very low (17%). Expression of LZ-EGFR-GFP stimulated strong BrdU incorporation without the requirement for EGF stimulation (42%). This suggests that LZ-EGFR-GFP functions similarly to EGF-activated EGFR-GFP in promoting cell proliferation. Furthermore, to demonstrate that the strong BrdU incorporation is indeed due to the expression and kinase activity of LZ-EGFR-GFP, we treated cells with AG1478 to block the specific EGFR tyrosine kinase activity of LZ-EGFR-GFP. We showed that AG1478 reduced BrdU incorporation level to 9% in cells expressing LZ-EGFR-GFP and to 11% in EGF-stimulated cells expressing EGFR-GFP (Figure 8). Together, our results indicate that expression of LZ-EGFR-GFP stimulates cell proliferation in a manner similar to EGF-activated EGFR-GFP. These results strongly suggest dimerization of LZ-EGFR and its subsequent activation is sufficient to cause a physiological outcome such as cell mitogenesis.

## 3. Discussion

The binding of EGF to EGFR results in the receptor dimerization, intrinsic kinase activation, C-terminal autophosphorylation, and the association with downstream signalling proteins. The early events following ligand binding are essential for EGFR to initiate cell signalling cascades, and for its endocytosis and routing to the lysosome for degradation [1]. However, it is not known whether ligand binding directly controls all of these post-binding events or whether ligand binding only controls dimerization of the receptors, while ligand-independent receptor dimerization controls EGFR kinase activation, autophosphorylation, and binding to downstream proteins. The objective of this study was to determine whether EGFR dimerization itself is sufficient to fully activate EGFR, stimulate various signalling pathways, and cause its endocytosis.

To achieve this objective, we first established a system to allow EGFR to dimerize in the absence of ligand. As demonstrated in this study, we accomplished this through swapping in a LZ in place of the native extracellular domain of EGFR. Previous studies have shown that high affinity dimers form when two complimentary zippers are in close proximity to one another [17]. It was reported that the replacement of the entire extracellular domain of the growth hormone receptor (GHR) by LZ of c-Jun or c-Fos resulted in the forced dimerization of GHR. The dimerization leads to the constitutive activation of various GHR signalling pathways [18]. Using a similar approach, we fused the c-Fos LZ to the transmembrane and cytoplasmic domains of EGFR. We showed that LZ-EGFR-GFP was constitutively dimerized when transiently expressed in 293T cells (Figure 2). The dimer induced by the LZ is very stable, as we frequently observed dimerized LZ-EGFR-GFP even after SDS-PAGE under reducing conditions (Figure 5). Similar phenomena have been observed for LZ-fused growth hormone receptors [18]. A strong and stable dimerization induced by LZs provides a good model to study the role of non-ligand induced dimerization on EGFR-mediated signalling and endocytosis.

By using this system, we first determined whether LZ-induced dimerization of EGFR is able to activate EGFR kinase activity and result in the autophosphorylation of EGFR C-terminal tyrosine residues. We showed that LZ-EGFR-GFP was strongly phosphorylated and this phosphorylation is dependent on the intrinsic kinase activity of EGFR (Figure 4). Moreover, the phosphorylation pattern of LZ-EGFR-GFP is very similar to that of EGF-stimulated EGFR. It is well established that EGF stimulates the phosphorylation of five major tyrosine residues at the EGFR C-terminus, including Y992, Y1068, Y1086, Y1148 and Y1173. We showed that all five tyrosine residues were phosphorylated in LZ-EGFR-GFP (Figure 6). Together, these results suggest that LZ-induced dimerization of EGFR activates EGFR kinase activity, resulting in the phosphorylation of the EGFR C-terminus to the same extent as that induced by EGF.

We next determined whether LZ-induced dimerization of EGFR stimulates EGFR endocytosis and EGFR-mediated cell signalling. We showed, using both fluorescence microscopy and subcellular fractionation, that constitutive dimerization of EGFR by the LZ leads to the receptor’s internalization into endosomes in the absence of EGF (Figure 3 and Figure 4). Moreover, LZ-EGFR-GFP remains phosphorylated at both the plasma membrane and endosomes (Figure 4). Endocytosis of EGFR can therefore be achieved in the absence of ligand as long as it is dimerized, as we have demonstrated previously [19].

We have also shown that the observed constitutive activation of LZ-EGFR-GFP is indeed due to LZ-induced dimerization, rather than the deletion of the extracellular domain of EGFR (Figure 5).

We further showed that constitutively active LZ-EGFR-GFP is able to stimulate many signalling proteins including SHC, PLC-γ1, Erk and Akt (Figure 7). Moreover, expression of LZ-EGFR-GFP in 293T cells induced cell proliferation in the absence of serum or EGF (Figure 8). This shows that LZ-induced dimerization of EGFR alone is sufficient to activate EGFR, induce EGFR endocytosis, stimulate various signalling pathways, and eventually cause cell proliferation.

While the phosphorylation level of LZ-EGFR-GFP is similar to that of EGFR-GFP following EGF stimulation, LZ-EGFR-GFP activates downstream signalling proteins and stimulates cell proliferation to a lower extent. The diminished potency of LZ-EGFR-GFP signalling outcomes may be attributable to a couple factors. First, LZ-EGFR-GFP is constitutively endocytosed and targeted to lysosomes for degradation, which may result in the quick termination of a substantial fraction of LZ-EGFR-GFP-mediated signalling. Second, downstream signalling proteins are being constantly activated by LZ-EGFR-GFP, and thus activation levels of these signalling proteins will likely be reduced with time. Therefore, it is very reasonable to observe sustained but low levels of signalling protein activation in cells transfected with LZ-EGFR-GFP. A third factor is that the truncation and substitution of the EGFR extracellular domain may result in the slight conformation change of LZ-EGFR-GFP and consequentially a reduction in the receptor’s stimulatory potency.

It is intriguing to compare LZ-EGFR-GFP with oncogenic ErbB2. It has been reported that oncogenic ErbB2 possessing a single mutation at its transmembrane domain (V664E) is constitutively dimerized and permanently active [8]. Like EGFR, this activated ErbB2 homodimer is internalized rapidly, though independent of ligand [7,10]. The constitutively activated ErbB2 also binds various downstream signalling proteins including Grb2 and Shc [20]. Similar to LZ-EGFR-GFP, ligand binding is not required for the activation, trafficking and signalling of oncogenic ErbB2, suggesting that the mutation-induced dimerization may be the principle driving force behind its activation, trafficking, and signalling potential. Other evidence supporting a role of dimerization in the activation of receptors comes from studies on a type-III deletion variant of the EGFR (EGFRvIII). EGFRvIII is devoid of amino acids 6–273, which spans the receptors EGF binding domain. Consequently, EGFRvIII is constitutively active and dimerized [21], which implies that dimerization is the key driving force for the activation of EGFRvIII. In other studies, leucine-zipper-induced dimerization of human growth hormone receptor (GHR) also leads to full activation of receptor in the absence of ligand [18]. All of these results lean towards the importance of receptor dimerization, rather than ligand binding, in receptor activation, cell signal initiation, and receptor trafficking.

Various ligands including EGF and TGF-α are able to dimerize EGFR and activate EGFR tyrosine kinase activity; however, they result in modulated binding affinities to downstream proteins and different rates of EGFR endocytosis. These results may suggest that ligand binding is not only required for receptor dimerization, but also determines certain signalling outcomes following dimerization. For example, signals derived from TGF-α binding to EGFR will not lead to cell proliferation via EGFR, while EGF is sufficient to cause cell proliferation [22]. A possible explanation for the different effects of EGF and TGF-α may be different dimer stabilities affected by these two ligands. Indeed, it has been shown that binding between TGF-α and EGFR is less resistant to low pH than binding between EGF and EGFR. In early endosomes, the EGFR-TGF-α complex dissociates and EGFR recycles back to the plasma membrane [22]. It is very likely that the TGF-α-induced EGFR dimer dissociates following the uncoupling of TGF-α from EGFR; dissociation of the EGFR dimer then results in the altered trafficking and interactions with downstream signalling proteins.

We have shown previously that EGFR dimerization is necessary to stimulate EGFR internalization [13]. Our results with LZ-EGFR-GFP clearly demonstrate that the dimerization of EGFR in the absence of ligand is also sufficient to activate EGFR, stimulate its endocytosis, and effect signalling.

## 4. Materials and Methods

### 4.1. Antibodies and Chemicals

Rabbit polyclonal antibodies to EGFR, ERK, phosphor Akt, and Shc were purchased from Santa Cruz Biotech (Santa Cruz, CA, USA). Mouse monoclonal antibody to phospho-EGFR was from Upstate Biotechnology Inc. (Lake Placid, NY, USA). Mouse anti-phospho-PLC-γ1 antibody was from Medicore (Montreal, QC, Canada). Mouse anti-EEA1 antibodies were from BD Signal Transduction (San Jose, CA, USA). AG1478 and monensin were from Calbiochem (La Jolla, CA, USA). EGF was from Upstate Biotechnology. Unless otherwise specified, all the chemicals were purchased from Sigma (Oakville, ON, Canada).

### 4.2. The Plasmids

The chimeric EGFR-GFP vector was engineered by inserting in frame the full-length EGFR into the pEGFP-N3 mammalian expression vector (Clontech, Palo Alto, CA, USA). A XhoI site and a KpnI site were introduce into the 5′ end and 3′ end of the full length EGFR by polymerase chain reaction (PCR), respectively. The fragment was then ligated and inserted in frame into the pEGFP-N3 mammalian expression vector. The chimeric LZ-EGFR-GFP receptor was engineered by joining the EGFR signal sequence (corresponding to amino acids-24-1 according to [23]) to the c-Fos LZ domain (corresponding to amino acids 160–200 according to author of [24]), followed by the transmembrane and intracellular domain of EGFR (corresponding to 623–1210 residues according to the authors of [23]). An XhoI site was introduce into the 5′ end and a HindIII site was introduced into the 3′ end of the EGFR signal sequence. A HindIII site and a SalI site were introduced into the c-Fos LZ 5′ end and the 3′ end by PCR, respectively. Similarly, a SalI site and a KpnI site were introduced into the 5′ end the 3′ end of the EGFR transmembrane and intracellular domain by PCR, respectively. Purified EGFR signal sequence, c-Fos LZ domain and the EGFR transmembrane and intracellular membrane domain fragments were then ligated and inserted in frame into the pEGFP-N3 vector (Clontech Laboratories, Palo Alto, CA, USA) (Figure 1). Sequence of this construct indicates the presence of EGFR leading sequence, LZ, some restriction sites and the EGFR transmembrane and intracellular domain followed by GFP.

As a control, we made another construct by deleting LZ from LZ-EGFR-GFP. We termed this construct as ΔED-EGFR-GFP (Figure 1). To make ΔED-EGFR-GFP, the 3′ end SalI site of transmembrane and intracellular domain of LZ-EGFR-GFP was mutated to HindIII by PCR. Purified EGFR transmembrane and intracellular domain was then ligated with EGFR signal sequence and pEGFP-N3 vector that were excised directly from LZ-EGFR-GFP using KpnI and HindIII. The sequence of DNT-EGFR-GFP was proved by DNA sequencing. The chimeric DsRed-Rab5 vector was engineered by inserting in frame the complete Rab5 into the pDsRed-C1 mammalian expression vector (Clontech, Palo Alto, CA, USA).

### 4.3. Cell Culture and Treatment

Human embryonic kidney 293T cells were cultured at 37 °C with Dulbecco’s Modified Eagle’s Medium (DMEM) with 10% foetal bovine serum (FBS) in a 5% CO_2_ atmosphere. To activate the transiently expressed chimeras, transfected cells were serum starved for 24 h. EGF was then added to a final concentration of 100 ng/mL for 30 min. For cells treated with EGFR specific tyrosine kinase inhibitor AG1478, cells transiently expressing for 48 h were treated with 0.5 µM AG1478 for 2 h and EGF was added accordingly to a final concentration of 100 ng/mL in the last 30 min. Transient transfection was carried out by calcium phosphate precipitation.

### 4.4. Subcellular Fractionation and Total Cell Lysates

Subcellular fractionation was conducted by the method described previously [25]. Briefly, following treatment cells were scraped and homogenized inhomogenization buffer (0.25 M sucrose, 20 mM Tris-HCl, pH 7, 1 mM MgCl_2_, 0.5 mM Na_3_VO_4_, 0.02% NaN_3_, 0.1 mM 4-(2-aminoethyl)-benzenesulfonyl fluoride, 4 mM NaF, 10 g/mL aprotinin, 1 µM pepstatin A). The homogenates were centrifuged at 200× *g* for 5 min to remove nuclei and other cell debris (P1). Then, the post-nuclear supernatant (S1) was centrifuged for 10 min at 1500× *g* to generate a supernatant (S2) and a pellet (P2). Afterwards, P2 was redissolved in homogenization buffer and overlaid with an equal volume of 1.42 M sucrose buffer. Following the centrifugation at 82,000× *g* for 1 h the pellicule at the interface of 0.25–1.42 M was collected as the plasma membrane (PM) fraction. With further centrifugation (100,000× *g* for 30 min) of the S2 fraction, a soluble CY fraction and a microsomal pellet were produced. The resulting pellet was resuspended in 0.25 M sucrose buffer and overlaid on top of a discontinuous sucrose gradient containing equal volumes of 1.00 and 1.15 M sucrose in homogenization buffer. After centrifugation at 200,000× *g* for 1.5 h, an EN fraction at the 0.25–1.00 M interface was collected. For a typical experiment, the total yielding is 30 µg for the plasma membrane, 30 g for the EN fraction and 1 mg for the cytosol fraction. The yielding of each fraction was quite consistent under all of the treatments.

For the total cell lysates, transiently expressing cells were lysed with 0.4% Triton X-100 lysis buffer (0.4% triton X-100, 140 mM NaCl, 50 mM Tris-Cl, pH 7.2, 1 mM EGTA) in the presence of protease inhibitors (0.1 mM 4-(2-aminoethyl)-benzenesulfonyl fluoride, 10 µg/mL aprotinin, 1 µM pepstatin A) for 1 h at 4 °C. Lysates were then cleared by subjection to centrifugation at 20,000× *g* for 30 min. The supernatant was then boiled in SDS-loading buffer (250 mM Tris-Cl, 40% glycerol, 8% sodium dodecyl sulfate, 20% β-mercaptoethanol, 2% bromophenol blue) at 95 °C for 5 min.

### 4.5. Immunoblotting

Protein samples were separated by SDS-PAGE and then transferred onto nitrocellulose membranes (BioRad, Hercules, CA, USA) electrophoretically by a semi-dry blotting apparatus at 15 mA per minigel for 45 min in transfer buffer. Membranes were then probed with the various primary antibody followed by respective horseradish peroxidase (HRP)-conjugated secondary antibody. The protein bands were detected by enhanced chemiluminescence and exposure to X-ray film.

### 4.6. Dimerization Assay

293T cells were harvested and pelleted following treatment. Cell pellets were resuspended in PBS in the presence of 0.5 mM Na_3_VO_4_, 0.02% NaN_3_, 0.1 mM AEBSF, 10 µg/mL aprotinin, 1 µM pepstatin A. Resuspensions were then homogenized in a glass homogenizer and collected. To these homogenates the crosslinker, Disulfosuccinimidyl suberate (DSS), was added to a final concentration of 6 mM. The mixture was then incubated at room temperature for 30 min after which the reaction was quenched with 250 mM glycine for an additional 15 min at room temperature. The treated homogenate was then subjected to ultra centrifugation at 100,000× *g* for 1 h. The pellet collected was then lysed in 0.4% Triton X-100 lysis buffer as described above overnight at 4 °C. Lysates were then cleared by subjection to centrifugation at 20,000× *g* for 30 min. The supernatant was then boiled in 4× SDS-loading buffer at 95 °C for 5 min prior to SDS-PAGE.

### 4.7. Fluorescence Microscopy

293T cells were seeded on glass coverslips. At 70% confluency, the cells were serum starved for 24 h. Following various treatment, the cells were fixed by methanol of −20 °C. To detect EGFR-GFP and LZ-EGFR-GFP alone, fluorescence excitation of the GFP tag was visualized with a Zeiss, Axiovert 200 fluorescent microscope (Zeiss Germany, Oberkochen, Germany). Co-localization of the GFP tagged chimera with a DsRed tagged Rab5 was done following the co-transfection of both fluorescent tag-encoding vectors into 293T cells. To stain pEGFR, cells were incubated with anti-pEGFR antibody at room temperature for 1 h followed by TRITC-conjugated secondary antibody for 1 h.

### 4.8. Bromodeoxyuridine (BrdU) Incorporation Assay

DNA synthesis was examined by bromodeoxyuridine (BrdU) incorporation. 293T cells were plated upon glass coverslips and transiently transfected with the chimeric constructs. Following expression for 48 h, cells were washed three times with PBS and serum starved for 24 h. Cells were then treated with EGF and/orAG1478 for 16 h. After incubation with BrdU (25 µM) for 8 h, cells were washed and fixed. DNA was denatured with 2 N HCl for 30 min at room temperature. To stain BrdU cells were incubated with mouse antibody to BrdU for 1 h followed by FITC-conjugated secondary antibody for 1 h. Total DNA was stained by propidium iodide (50 µg/mL). The percentage of cells with positive DNA synthesis was calculated as the ratio between the number of BrdU positive cells and the total number of cells (propidium iodide positive) × 100. For each experimental treatment, a minimum of 300 cells were counted.

## 5. Conclusions

In conclusion, we show that substitution of the complete extracellular domain with a c-fos LZ domain results in the dimerization of EGFR. This non-ligand-induced dimerization of EGFR results in the constitutive activation of EGFR tyrosine kinase and phosphorylation at the five major tyrosine residues in the C-terminus. Constitutively activated LZ-EGFR-GFP is also constitutively endocytosed into endosomes and is able to activate several signalling pathways, including those leading to the stimulation of cell proliferation. We conclude that receptor dimerization is a critical event in EGFR activation, trafficking, and subsequent downstream signalling.
